# Exploring the Relationship Between Electronic Device Use and Psychological Dimensions of Procrastination in University Students

**DOI:** 10.3390/bs16010006

**Published:** 2025-12-19

**Authors:** María Salguero-Pazos, Salvador Reyes-de-Cózar

**Affiliations:** Department of Communication and Education, Universidad Loyola Andalucía, 41704 Dos Hermanas, Spain; sreyes@uloyola.es

**Keywords:** electronic devices, screen use, academic procrastination, university students

## Abstract

This study examines the relationship between electronic device use and psychological dimensions of procrastination in university students. The main objectives were to identify screen usage habits, explore their association with key psychological factors, and determine whether these factors predict perceived procrastination. An ad hoc instrument and descriptive, correlational, and regression techniques were used for data analysis of a sample of 924 Spanish university students. From this sample, a target subsample of 386 students who reported high levels of procrastination was selected to analyse screen-use patterns and their links with procrastination dimensions. In contrast, the full sample was used to estimate the predictive regression models. Results showed high screen use among students, with 20% potentially at risk for problematic use. High screen use correlated with lower self-regulation, self-efficacy, and self-esteem, and higher anxiety. Conversely, higher self-regulation and self-efficacy were linked to more motivation, better self-esteem, and less anxiety. Regression results indicated that screen time, self-regulation, and self-efficacy significantly predict procrastination levels. These findings suggest that psychological factors play a key role in how screen use relates to procrastination.

## 1. Introduction

Electronic devices are now necessary in students’ daily lives in university environments. This increased use has raised concerns about their potential effects on students’ psychological well-being and academic performance. (Lee & Hancock, 2024; Twenge et al., 2018). Nowadays, young people spend more time interacting with these devices, which can lead to addiction to devices or social networks (Brailovskaia et al., 2021; Lee & Hancock, 2024). This addiction is characterized by emotional dependence and a feeling of discomfort when use is interrupted, significantly disrupting students’ daily routines and lifestyles and sometimes leading to personal and social problems (Marco & Chóliz, 2013). The obsession with social networks leads to students spending long hours online, neglecting other fundamental areas of their lives such as studies, work, family, or friends (Castro Bolaños & Mahamud Rodríguez, 2017). This lack of control over the use of electronic devices may result in poor academic performance, as students may experience difficulties concentrating and fulfilling their academic responsibilities. In addition, addiction to social networking may predispose students to social isolation and disconnection from reality, contributing to the manifestation of depressive symptoms and low self-esteem (Awopetu et al., 2024; Sujarwoto et al., 2023). These emotional problems can be intensified by emotional instability, irritability, and lower frustration tolerance, further affecting students’ overall well-being and mental health (Núñez-Guzmán & Cisneros-Chavez, 2019). Constant use of social networks can lead students to experience emotional dependence and feelings of discomfort when they are not online, negatively affecting their daily routines and overall well-being (Marco & Chóliz, 2013). This disconnection from fundamental aspects of life can lead to a lack of fulfillment of academic responsibilities, poor academic performance, and other problems in school settings.

Procrastination is another risk factor that can lead to poor academic performance and school problems in students, and it is associated with demotivation and the intention to drop out. Procrastination is a widely observed behavior among university students that generates serious drawbacks in their academic performance, significantly hindering the teaching-learning process (Hayat et al., 2020; Rezaei-Gazki et al., 2024). This behavior, which is very common in the general population, not only hurts the effectiveness of learning methods but also has a considerable impact on levels of personal well-being, causing states of anxiety or depression (Amarnath et al., 2023; Bela et al., 2023; Ihkamuddin et al., 2024). This behaviour is described as postponing or deferring an activity until later, substituting it for a less important activity (Steel, 2007). In academic environments, this results in students not handing in assignments on time or failing to meet teacher deadlines (Ramírez et al., 2020). Procrastination creates a discrepancy between intentions and actions, which significantly increases the likelihood of students experiencing negative emotions (Dardara & Al-Makhalid, 2022; Elizondo et al., 2024; Steel & Klingsieck, 2016). This mismatch can cause students to face various unpleasant emotions, such as anxiety about assessments, constant stress due to pending assignments, burnout, and even a loss of interest in academic activities, decreasing their motivation and commitment to study (Pereira & Ramos, 2021; Valente et al., 2024). These factors create an emotionally challenging environment that can hinder students’ academic performance and well-being. Accordingly, the study by Tantalean Terrones et al. (2024) shows that procrastination is frequently associated with anxiety, stress, low self-esteem, or self-efficacy, leading to negative grades and even school dropout due to students’ discomfort when they do not achieve their goals.

Procrastination is a complex phenomenon to which many factors are associated, depending on different approaches and authors and not always complementary, making it difficult to deepen the understanding of a critical phenomenon from an educational point of view. Thus, many researchers have linked procrastination to a failure in self-regulation (Elizondo et al., 2024; Steel & Klingsieck, 2016). However, others do not limit themselves to seeing it only as a problem of time management by students but also consider cognitive, affective, and behavioral aspects, such as overconfidence in trying to achieve a goal on time and behaviors related to self-efficacy (Moreta-Herrera et al., 2018). In addition, procrastination is associated with strategies to manage negative emotions, such as fear of failure, that leads students to postpone essential and complex tasks, preferring activities that offer an immediate reward and generate a temporary sense of well-being (Hen & Goroshit, 2020; Q. Wang et al., 2022). Recently, a study by Salguero-Pazos and Reyes-de-Cózar (2023) reviews models of procrastination by exploring their dimensionality along three axes: personal (authoritarian parenting), pedagogical (academic performance and student dropout), and psychological (self-regulation, self-efficacy, self-esteem, motivation, personality, and anxiety).

Based on the study by Salguero-Pazos and Reyes-de-Cózar (2023), this paper studies academic procrastination through the study of its dimensions that make up the psychological axis and directly affect procrastination in students. These are self-regulation, self-efficacy, personality, anxiety, self-esteem, and motivation. Self-regulation is related to the management of learning processes, where failures between intention and action increase the likelihood of procrastination and negative feelings (Lonka et al., 2014; Pereira & Ramos, 2021). Similarly, self-efficacy is a key predictor of academic success (Alzabidi et al., 2024), as higher levels enable students to face difficulties with confidence and adopt proactive study behaviors (Li et al., 2019; H. Wang et al., 2023), being directly linked to procrastination (Purnomo et al., 2024). Personality also influences academic behaviors, as several traits are related to procrastination (Cruz & Miranda, 2024) and academic burnout (Y. Hou, 2024), with studies showing significant correlations (Y. M. Hou et al., 2024; Liu & Jin, 2018). Likewise, anxiety is identified as an important factor in academic procrastination (Rezaei-Gazki et al., 2024; Saplavska & Jerkunkova, 2018), even considered a consequence of it (Ferrari et al., 1995), and is associated with depression, low academic performance, and school dropout (Karaman & Watson, 2017; Pascoe et al., 2020). Regarding self-esteem, procrastination generates feelings of guilt that negatively affect this dimension (Desai et al., 2021; Ghasempour et al., 2024). There is a relationship between high anxiety, low self-esteem, and procrastination (Parwez et al., 2023), with self-esteem being a determining factor in academic procrastination (Babu et al., 2019; Yang et al., 2023). Students with low self-esteem tend to excessively use social networks, promoting the postponement of tasks and affecting their well-being (Rodríguez & Moreno, 2019). Finally, motivation is fundamental for effective learning (Kemal et al., 2023); its absence is associated with higher levels of procrastination (Anggoro, 2021), highlighting the importance of fostering internal motivation to improve academic performance (Tisocco & Liporace, 2023). These data show that procrastination is a multidimensional construct influenced by multiple factors. Each of these dimensions contributes significantly to explaining the phenomenon, showing that procrastination cannot be understood as an isolated behavior but as the result of the complex interaction between emotional, cognitive, and behavioral variables.

The increased use of electronic devices among students has made the relationship between technology and procrastination significantly relevant in educational and psychological research. Numerous studies have addressed this phenomenon and have examined how excessive use of technology can influence students’ tendency to procrastinate on essential tasks (Geng et al., 2018; Rozgonjuk et al., 2018). Several studies have corroborated this significant association between the problematic use of electronic devices and procrastination. For example, research by Günlü and Ceyhan (2017) and Yurdakoş and Biçer (2019) found a positive correlation between these two factors, highlighting how technological dependence affects students’ ability to manage their time correctly and fulfill their academic responsibilities effectively. A line of research also focuses on the impact of constant connectivity and the habit of frequently checking one’s mobile phone. These studies suggest that these practices not only interfere with concentration on academic or work activities but also increase the likelihood of procrastination (Bayer et al., 2016; Vanden Abeele, 2021). These studies show how permanent connectivity, due to the use of screens and social networks, disrupts students’ attention, hindering their ability to stay focused on tasks unrelated to electronic devices, such as studying or working and causing individuals to procrastinate more frequently. Furthermore, Türel and Dokumaci (2022) reports that students who exhibit problematic behavior about using electronic devices are more likely to procrastinate. Moreover, this behavior directly affects their academic performance, as they spend a large part of their time using electronic devices, neglect their academic obligations, procrastinate on assignments, and often fail to complete their activities on time. Therefore, according to these data, procrastination could be a consequence of the excessive use of electronic devices. This behavior also affects students’ academic performance and can hurt their well-being (Meier, 2022). As dependence on technology increases, it is crucial to recognize its adverse effects on students’ time management, productivity, and mental health. It is imperative to continue researching and developing strategies to mitigate these detrimental effects.

Based on the above, it is essential to empirically explore the connections between screen use in educational settings and how it is associated with the dimensions that make up procrastination. Both intensive screen use and the tendency to procrastinate have been established as determining predictors of academic failure and emotional distress. Although the scientific literature has documented the relationship between the use of electronic devices and academic procrastination, most previous studies have addressed procrastination as a single construct. The primary contribution of this research lies in analysing procrastination in its psychological dimensions. Instead of evaluating the phenomenon as a whole, this study examines how screen use is associated explicitly with components such as self-regulation, anxiety, and motivation. This multidimensional approach enables a more rigorous understanding of the psychological mechanisms that link technology to academic behaviour. From an academic point of view, this understanding is critical to developing effective strategies and actions that help students manage their time better and reduce procrastination induced by the excessive use of electronic devices, thus helping to improve academic performance. It is for this purpose that the following research questions are proposed for this study:

- RQ1: What are the predominant screen usage habits among university students?
- RQ2: How is screen use related to the dimensions of procrastination?
- RQ3: Is it possible to predict students’ perceived level of procrastination by knowing the dimensions associated with procrastination?

## 2. Materials and Methods

### 2.1. Research Method

This study, based on the theoretical framework proposed by Salguero-Pazos and Reyes-de-Cózar (2023) and the psychological dimensions highlighted in this study directly related to procrastination, developed an instrument to measure the relationship between screen use and the psychological dimensions of procrastination in the literature. The instrument has been created using the adaptation of different validated scales that address the different dimensions linked to procrastination and screen use.

The selected sample was analyzed using statistical techniques to identify the study’s objectives. Descriptive statistics were used to determine the state of the sample about the different psychological dimensions of procrastination, as well as frequencies to determine the screen use habits of the students. Correlation techniques, exploratory factor analysis, and linear regressions were also used to determine the relationships between the different variables of the study, their meaning and strength, and which factors or dimensions of the study can predict procrastination in university students.

### 2.2. Sample

The sample was selected using a non-probabilistic convenience sampling technique, primarily based on the accessibility and availability of participants within the university context. The research team approached students directly in their classrooms following authorisation from the faculty. This face-to-face recruitment ensured that participants were active students attending regular academic sessions. Regarding the data collection instrument, the questionnaire was designed and administered through the Qualtrics XM online platform (https://www.qualtrics.com/, accessed on 18 December 2025), which allowed for secure data storage and a standardised presentation of the items. All items included in the questionnaire were mandatory; therefore, it was not necessary to treat missing values. Cases that were incomplete due to the questionnaire not being completed were discarded.

The sample consisted of 924 Spanish university students distributed among the five areas of knowledge. The study employed a two-step sampling strategy aligned with the research questions. The initial convenience sample consisted of 924 Spanish university students (sample 1), which was used to estimate the predictive regression models (RQ3). To address RQ1 and RQ2, which focus specifically on screen-use patterns and their association with procrastination, a subsample of 386 students (sample 2) was selected from the initial pool. This subsample (sample 2) was defined through a screening item on perceived frequency of academic procrastination; only students reporting high levels (“Almost always” or “Always”) were included to ensure that these analyses focused on individuals with clearly problematic procrastination behavior. (Table 1).

### 2.3. Instrument

For this study, an ad hoc instrument was developed that measures screen use habits and procrastination broken down into the psychological dimensions associated with the construct following the procrastination framework proposed by Salguero-Pazos and Reyes-de-Cózar (2023): Self-regulation, Self-efficacy, Motivation, Self-esteem, Personality and Anxiety.

The questionnaire items were adapted from previously validated instruments that have demonstrated adequate validity and reliability, showing good internal consistency (Cronbach’s α between 0.70 and 0.90). The Self-regulation dimension, understood as the ability to control and manage thoughts, emotions, and actions through a series of personal strategies to achieve goals and avoid undesired results, an adaptation of the Academic Self-regulation Scale (ASRS) (Akhtar & Mahmood, 2013) and the Emotion and Motivation Self-regulation Questionnaire (EMSR-Q) (Alonso-Tapia et al., 2014) was established to assess this variable. The Student Self-Efficacy Scale (SSE) (Rowbotham & Schmitz, 2013) measures the Self-Efficacy dimension, defined as the personal perception or belief of one’s capabilities for a given situation. The Motivation variable, a set of internal factors that can determine a person’s actions in academic settings, is measured with the Academic Motivation Scale-College (AMS-C) (Vallerand et al., 1992). The Rosenberg Self-Esteem Scale (RSES) (Rosenberg, 1965) assessed students’ Self-Esteem, referring to the sets of perceptions, thoughts, evaluations, and feelings directed towards oneself. The International Personality Item Pool (IPIP) (Goldberg et al., 2006) was used to measure Personality, understood as the psychic characteristics of a person that determine how he/she acts in certain circumstances. For the Anxiety dimension, defined as a mental state characterized by great restlessness, intense excitement, and extreme insecurity, a scale combining the Text Anxiety Inventory (TAI-5 item) (Taylor & Deane, 2002) has been established to assess anxiety in academic settings focusing on students’ test anxiety, with The State-Trait Anxiety Inventory (STAI-T) (Spielberger, 1970) which measures state anxiety.

For the evaluation of the use of electronic devices, the instrument created is based on the selection of ten items from the Adolescent Preoccupation with Screen Scale (APSS) (Hunter et al., 2017), which measures students’ screen addiction, screen time, or problematic screen use, and the adaptation of the nine-item version of the Social Media Disorder Scale (9-item SMD scale) (van den Eijnden et al., 2016), which measures addiction to social networks in particular and the possible effects of this.

The scales used to create the instrument are summarised in Table 2.

**Figure 1:**
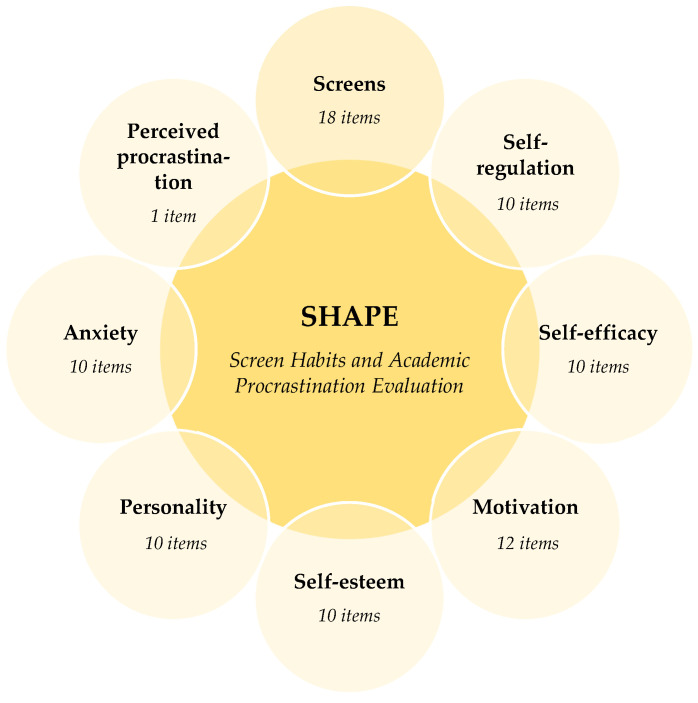
Graphical illustration of the instrument SHAPE.

The final instrument (SHAPE: Screen Habits and Academic Procrastination Evaluation) consists of Likert-type items ranging from 1 to 5, with 1 being the minimum value and 5 being the maximum. The items in the questionnaire respond to the question: To what extent do you agree or disagree with the following statements? Additionally, students were asked to indicate their perception of their level of procrastination. The question refers to their tendency to procrastinate when it comes to academic tasks. The answers are also based on a Likert scale ranging from those who never procrastinate (1) to those who always do (5). Thus, the final instrument consists of 81 items: 80 of which measure screen and social media use, as well as the psychological dimensions of procrastination included in the study, and one independent item that refers directly to students’ perception of their own procrastination (Figure 1). The questionnaire was introduced with an informed consent and information sheet to ensure all students understood the items. Throughout the process, students were accompanied by a researcher to clarify and resolve any doubts regarding their understanding of the items.

### 2.4. Data Analysis

Descriptive, correlational, and regression techniques are used to analyze the data obtained; for the descriptive analysis, frequencies and measures of central tendency and dispersion, such as mean, minimum, maximum, and standard deviation, are used. Pearson’s correlation coefficient was used to identify the possible relationships between the dimensions of procrastination that most influence screen use in university students and the strength and direction of these, as well as regression techniques to determine the dependence between the study variables. All calculations and statistical analyses were done using SPSS software, version 24.

## 3. Results

### 3.1. Characteristics of the Sample

The samples used in this study show a similar distribution in terms of gender, age, academic year, and area of knowledge. The total sample of the study, or sample 1, ranges in age from 17 to 41, with a mean age of 20. Likewise, 20 is the most represented age, with 20.2%, followed by 19, with 19.7% representation. The subsample or sample 2 has an age range between 17 and 41 years and an average age of 20. The age of 18 is the most represented at 18.9%, followed by 19 and 20 years, which represent 18.4% of the sample, respectively.

**Figure 2:**
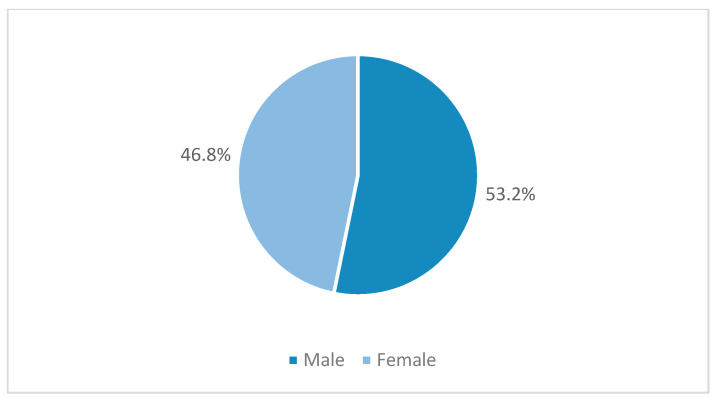
Distribution of sample 1 by gender.

The total sample or sample 1 of the study consists of 924 students, of whom 432 (46.8%) are female and 492 (53.2%) are male (Figure 2).

**Figure 3:**
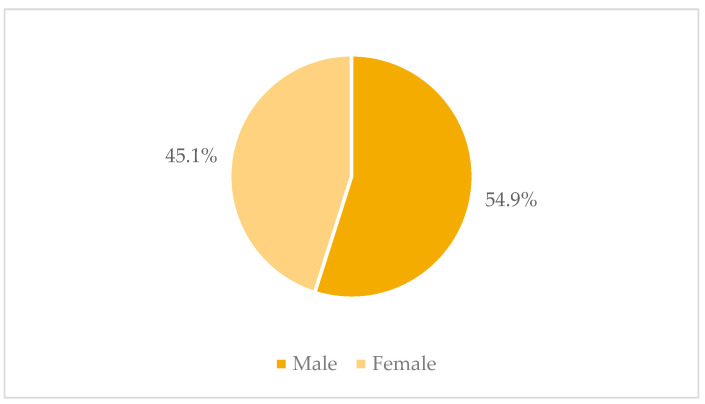
Distribution of sample 2 by gender.

The subsample or sample 2 of the study consists of 386 students, of whom 174 (45.1%) are women and 212 (54.9%) are men (Figure 3).

**Figure 4:**
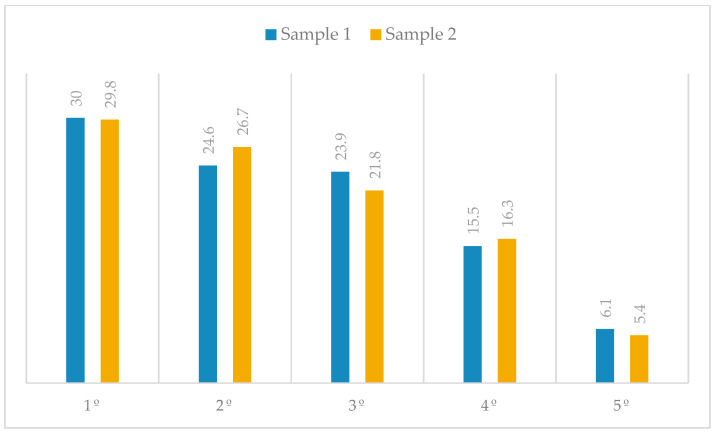
Distribution of percentages by academic year.

On the other hand, almost 80% of the sample consists of students in the first three years in both samples, with the first year being the most represented, at 30% in sample 1 and 29.8% in sample 2. In the fourth year, we observe a decrease in the sample, with a representation of 15.5% in sample 1 and 5.4% in the subsample (sample 2). In these classrooms, there were fewer students and, in turn, fewer willing to answer the questionnaire. The least represented year in both samples is the fifth, with 6.1% and 5.4% of the total, respectively. This data reflects the actual structure of Spanish degree programs, in which most degrees last four years and only a limited number of double degrees extend to a fifth year (Figure 4).

**Figure 5:**
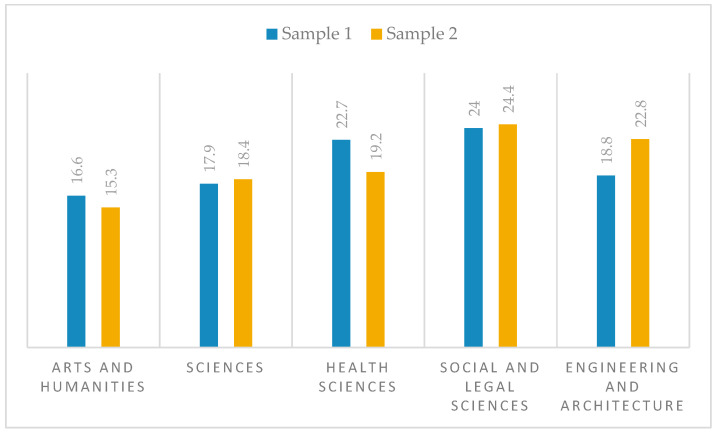
Distribution of percentages by areas of knowledge.

In terms of areas of knowledge, in both samples, the area of Social and Legal Sciences stands out as the most represented in the samples, with 24% in sample 1 and 24.4% in sample 2, compared to the Arts and Humanities area, which has the lowest representation, with 16.6% in sample 1 and 15.3% in sample 2. The percentages by area of knowledge for both samples are shown in Figure 5.

### 3.2. Internal Structure of the Instrument

The internal consistency of the instrument was calculated using Cronbach’s Alpha coefficient with a result of α = 0.852. The result of the internal consistency by scale factors is generally positive, except for the value for the personality and self-regulation dimensions, as shown in Table 3.

### 3.3. Screen Usage Habits of Students

**Figure 6:**
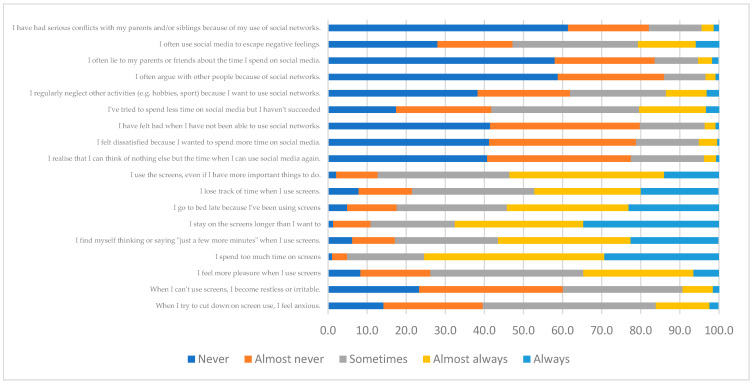
Distribution of sample 2 for the Screen Use dimension.

The results regarding the RQ1, which analyses the predominant screen use habits among university students, are shown. Respondents answered Never or Almost never in most of the items related to problematic behavior, for example, in the item ‘I often argue with other people because of social networks’ with 58.8% of young people answering Never and 27.2% Almost never or ‘I have had serious conflicts with my parents and siblings because of my use of social networks’ where 61.4% Never show this behavior and 20.7% Almost never. Moreover, in general terms, they show high use of screens, since items such as ‘I stay on screens longer than I want to’, with 32.9% of students answering Almost always and 34.7% recognizing that they always do so, as well as the item ‘I spend too much time on screens’ with results of 46.1% Almost always and 29.3% of students who always show this behavior (which represents 75.4% of the sample). Also, more than 50% of respondents admit that they sometimes or almost always try to spend less time on social networks but fail and but fail, and they go to bed late because they have been using screens (Figure 6).

### 3.4. Psychological Dimensions of Procrastination

For each dimension extracted from the framework linked to procrastination (Self-regulation, Self-efficacy, Motivation, Self-esteem, and Anxiety), the descriptive statistical results obtained are shown in Table 4.

**Figure 7:**
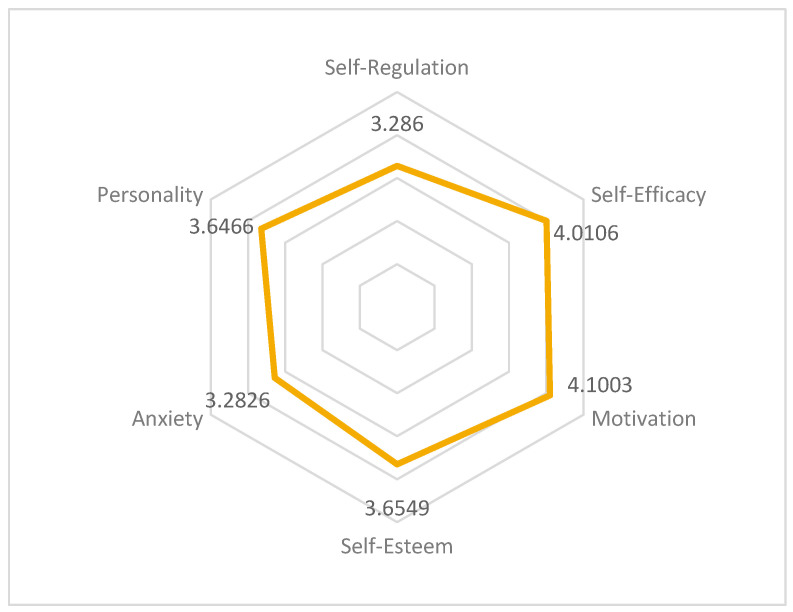
Procrastination dimensions mean.

According to the results, motivation is the variable with the highest mean, with a value of 4.10, presenting a maximum value of 5 and a minimum value of 1.91, the highest among all the scales. In second place, Self-efficacy, with a mean value of 4.01, is the second highest, with values ranging from 1 to 5. Self-esteem has a mean value of 3.654, with scores ranging from 1.60 to 5. Personality shows scores ranging from 1 to 4.9 with the third lowest average value (3.6466). Self-regulation stands out for having the lowest maximum value among the scales analyzed (4.8, compared to the value of 5 of the other dimensions) and a mean value of 3.286, the second lowest. Finally, Anxiety has the lowest mean value of all, at 3.282, with scores ranging between 1 and 5, the same range of scores as self-efficacy. The means for each dimension are shown in Figure 7.

### 3.5. Relationship Between Screen Use and Academic Procrastination According to Its Dimensions

About the RQ2 of the study, which aims to analyze how screen use is related to the dimensions of procrastination, a correlational study of the variables was carried out to find out the possible relationships between them and the extent to which they do so. The results obtained are shown in Table 5.

As shown in Table 5, the analysis results reveal significant correlations between screen use and the variables of self-esteem, self-regulation, self-efficacy, and anxiety, all at the 0.01 significance level, indicating a strong and reliable relationship between the variables. Screen and social media use are negatively associated with self-esteem, self-regulation, and self-efficacy, suggesting that higher screen use is linked to lower levels in these dimensions. In contrast, the relationship between screen use and anxiety is positive, meaning that students who use electronic devices more frequently also exhibit higher levels of anxiety. These results indicate that screen use impacts most dimensions studied, affecting students’ procrastination.

On the other hand, the analysis also reveals significant correlations between the different dimensions of procrastination included in the study, all at the 0.01 significance level. The dimensions of self-efficacy and self-regulation show significant correlations with all the procrastination dimensions analyzed. Both are negatively correlated with anxiety, indicating that students with higher levels of self-regulation and self-efficacy tend to experience lower levels of anxiety. Furthermore, these variables are positively associated with self-esteem, motivation, and personality, suggesting that better self-regulation and self-efficacy are also linked to higher levels of self-esteem and motivation and aspects of personality. A significant positive correlation is also observed between self-regulation and self-efficacy, showing that students with good self-regulation strategies tend to have higher self-efficacy.

Additionally, a positive correlation is observed between personality and students’ motivation and anxiety, with personality being a determining factor for both motivation levels and anxiety control.

Finally, significant correlations are also found between self-esteem and the variables of motivation and anxiety: self-esteem is positively associated with motivation and negatively associated with anxiety, indicating that students with higher self-esteem also tend to show greater motivation and lower anxiety levels.

### 3.6. Predictive Ability of Study Variables on Perceived Procrastination

Responding to the RQ3 of the study, which seeks to explore whether it is possible to predict students’ perceived procrastination by knowing the dimensions that are associated with procrastination, first, an exploratory factor analysis was carried out to identify underlying factors that could explain the correlations between the measured variables. This procedure made it possible to delimit the different factors present in the scales used in the study, thus facilitating a more structured interpretation of the constructs assessed. Subsequently, a multiple linear regression was performed with the total sample of 924 students to obtain more precise results, using the previously identified factors as predictors. This approach made it possible to identify which factors significantly influence procrastination, quantifying the effect of each one on the procrastinating behavior of university students.

Before interpreting the regression coefficients, we examined whether the main assumptions of multiple linear regression were met. Linearity and homoscedasticity were assessed through the scatterplot of standardised residuals versus standardised predicted values, which showed no marked curvature or funnel-shaped pattern. Normality of residuals was evaluated using histograms and normal P–P plots and was considered acceptable. Multicollinearity was checked via Variance Inflation Factors (all VIF between 1.14 and 1.79), and the Durbin–Watson statistic (DW = 2.05) indicated independence of errors. Overall, no serious violations of the regression assumptions were detected.

The overall results obtained for the model are shown below in Table 6 and Table 7.

The data presented in Table 6 and Table 7 indicate that the regression model was statistically significant, F (11, 912) = 38.63, *p* < 0.001, explaining 31.8% of the variance in perceived procrastination (R = 0.564; R^2^ = 0.318; adjusted R^2^ = 0.310). The standard error of the estimate was 0.679, indicating an acceptable level of prediction error.

The results of the influence of variables on perceived procrastination are shown in Table 8 below.

Looking at the results in Table 8, variables with a significant influence on student’s perception of procrastination were identified (*p* < 0.05). Screen time use is positively associated with perceived procrastination, indicating that more screen time predicts procrastination. Positive self-regulation shows a significant negative relationship, indicating that students with better positive self-regulation strategies perceived less procrastination. Likewise, negative self-regulation is positively related to procrastination, indicating that students with ineffective self-regulation strategies increase their procrastination. Finally, self-efficacy stands out as the last dimension that significantly affects perceived procrastination, with a positive effect, with higher levels of self-efficacy associated with higher levels of procrastination. This finding might be counter-intuitive; further analysis is required to analyze these results. On the other hand, variables such as total motivation, self-esteem, personality, and anxiety did not show significant relationships with perceived procrastination in this model.

The regression equation for the model obtained would be:

Perceived_Procrastination = 2.744 + 0.302 (FScreens_TimeUse) − 0.484 (FSelfReg_Positiv) + 0.149 (FSelfReg_Negativ) + 0.151 (Self_Effic_Total)

**Figure 8:**
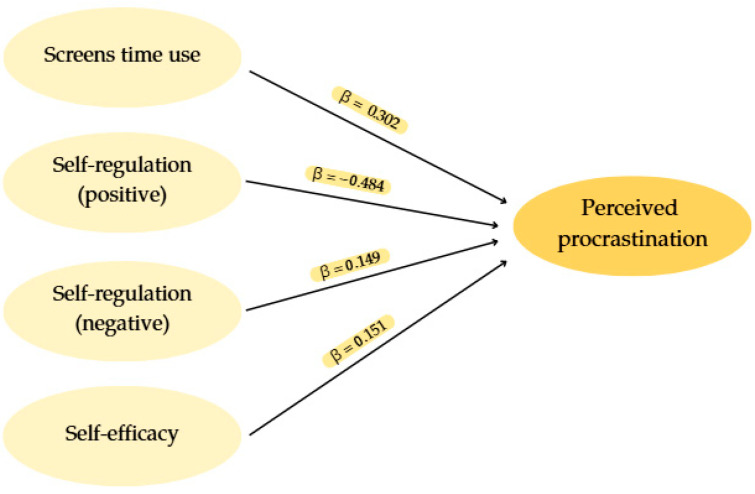
Graphical representation of the regression model.

Figure 8 illustrates the regression model. Screens time use, positive self-regulation, negative self-regulation and self-efficacy are represented as predictor variables, each pointing with a single-headed arrow towards perceived procrastination as the outcome variable. The arrows are labelled with the regression coefficients (β), indicating a positive association for screens time use, negative self-regulation and self-efficacy, and a negative association for positive self-regulation.

## 4. Discussion

Screens and electronic devices are now widespread, significantly transforming how university students interact with the world around them. While bringing numerous benefits, this transformation has posed considerable challenges, particularly in academic environments. The accessibility of social media and other forms of digital entertainment has led to increased procrastination, directly affecting students’ academic performance (Adelakun et al., 2023; Kutzhan et al., 2023).

The instrument (SHAPE: Screen Habit and Academic Procrastination Evaluation) has proven reliable in measuring screen use and procrastination through its psychological variables in university students. Internal consistency analyses confirm good overall reliability with a Cronbach’s Alpha of α = 0.852 for the instrument. The overall factor reliability results were positive, although the self-regulation and personality dimensions showed slightly lower internal consistency. Despite being lower values, these dimensions have Cronbach’s alpha values of 0.614 and 0.651, respectively, values that coincide with pilot studies in which Cronbach’s Alpha values between 0.6 and 0.7 are considered acceptable or with good internal consistency (Boyle, 2021; Ņikadimovs & Vēvere, 2024).

In relation to the first research question, which sought to identify the most prevalent screen usage habits among university students who participated in the study, the results obtained indicated that more than 75% admitted to spending too much time on screens. Almost 90% of the sample admit to “sometimes” to “always” using screens longer than they would like, even when they have more important things to do, causing them to lose track of time or go to bed late. Many studies refer to the increase in the use of social networks in recent years (Azzaakiyyah, 2023) and how university students spend a high proportion of their time on the Internet or social networks, triggering procrastination processes (Aznar Díaz et al., 2020). Although the data do not reveal generalized addictive behaviors, the need to be alert is highlighted, as items such as ‘I stay on screens longer than I would like’, ‘I spend too much time on screens’, ‘I go to bed late because I’ve been using screens’ and ‘I find myself thinking or saying “just a few more minutes” when I use screens’ obtained response percentages for the maximum value of the scale (Always) of 34.7%, 29.3%, 23.1% and 22.5%, respectively. Therefore, more than 20% of the students admit to always displaying this behavior in their daily lives, so, in other words, 2 out of 10 students could have a critical case of screen addiction.

The second research question of the study sought to analyse how screen use relates to the psychological dimensions of procrastination included in the analysis. To answer this question, a study was conducted to examine the correlations between these variables. The results indicated that among the psychological dimensions of procrastination, self-regulation, self-efficacy, and self-esteem are those that have shown a significant inverse correlation with screen use. Therefore, the fewer students use electronic devices or social media, the better their levels of self-regulation, self-efficacy, and self-esteem, which in turn reduces procrastination behaviours. This data is consistent with the findings of Castro Bolaños and Mahamud Rodríguez (2017), who found that more than 50% of the students surveyed demonstrated a lack of self-regulation when performing or fulfilling their academic commitments due to the excessive use of social networks. On the other hand, Lin et al. (2022) demonstrated in their study how electronic device addiction decreased self-efficacy in university students. Cheng et al. (2021) indicate that improving self-efficacy can mitigate device addiction levels among students, thereby reducing procrastination. Similarly, many studies relate self-esteem levels to the use of social networks and screens, showing in their results a negative correlation between the dimensions in which a higher level of addiction to electronic devices or social networks translates into a decrease in self-esteem levels (Smith et al., 2024; Subiyakto et al., 2024).

On the other hand, anxiety is found to have a significant direct correlation with students ‘screen use habits, which means that a high use of electronic devices increases students’ anxiety levels. Many studies relate high levels of stress, anxiety, or depression among individuals with social network addiction behaviors, having an impact on the quality of life of students who have this addiction (Favieri et al., 2024; Gao et al., 2022; Olivier et al., 2022). For this reason, these results seem to indicate that if we reduce the stress or anxiety levels among our students, we will help reduce screen addiction behaviors and procrastination habits, as well as improve their academic performance and quality of life.

The relationships between the psychological dimensions of procrastination and the interconnection between the different variables analyzed in the study are observed. Self-efficacy and self-regulation correlate with all the dimensions studied and between them. Therefore, they could be presented as critical variables to mitigate procrastination habits among university students. The dimensions of self-esteem, motivation, and personality show a direct correlation with self-efficacy and self-regulation, so students with higher levels of self-efficacy will also have good self-regulation, high self-esteem, higher levels of motivation, and a relationship with their personality traits. These studies are similar to those found in the literature, such as the study by Sabrina et al. (2023), which showed a significant positive correlation between self-efficacy and motivation and a positive correlation between self-regulation. Similarly, other studies found that self-efficacy significantly enhanced motivation, determining the level of success in accomplishing assigned tasks (Hasanah et al., 2019; Szczepańska-Gieracha & Mazurek, 2020). The study conducted by Usán Supervía et al. (2023) reveals that self-esteem significantly influences students’ self-efficacy and life satisfaction, underlining the importance of self-esteem for students’ personal and psychological development. These data show that improving students’ self-efficacy could translate into improved self-regulation with increased motivation and self-esteem and could contribute to a reduction in procrastination and improved academic outcomes and dropout rates of students. Self-efficacy and self-regulation also correlate directly with personality traits, measured in the big five personality traits of openness to experience, conscientiousness, extraversion, agreeableness, and neuroticism. This same result is shown in the study by (Furnham & Cheng, 2024), where four factors (extraversion, neuroticism, conscientiousness, and openness) were predictors of self-efficacy in the sample analyzed, which may help predict procrastination behaviors in students according to their personality traits.

On the other hand, the anxiety dimension presents an inverse correlation with self-efficacy and self-regulation, with students with lower levels of self-efficacy and self-regulation presenting more significant anxiety symptoms. Many studies have shown a negative correlation between self-efficacy and anxiety symptoms (Mousset et al., 2024; Roick & Ringeisen, 2017). Self-efficacy is considered a vital factor in coping with negative emotions in adolescents and improving their mental health, as those individuals with a high level of self-efficacy have low symptoms of depression or anxiety (Weurlander et al., 2024). Similarly, several studies also highlight self-regulation skills as a predictor and cause of different types of anxiety (Kalamazh et al., 2024). These data are consistent with those obtained in the study by Herawati et al. (2023), which demonstrate a negative relationship between students’ self-regulated learning and anxiety levels. These findings could suggest that enhancing self-regulation and self-efficacy skills may effectively reduce anxiety across various life stages and contexts.

Likewise, anxiety correlates negatively with students’ self-esteem, as in the Kumari (2024) study, which indicates that individuals with higher anxiety are more likely to have lower self-esteem. Thus, students with lower self-efficacy and lower self-regulation will have higher levels of anxiety, leading to lower self-esteem. Therefore, working on self-efficacy and self-regulation with students is not only interesting in terms of improving procrastination but also focuses on their mental health, helping to improve self-esteem and reduce anxiety.

In addition, self-esteem presents a positive or direct correlation with motivation levels, with students with better self-esteem data being more motivated to perform their academic tasks. These results coincide with research demonstrating the positive correlation between both dimensions and how they help improve student learning (Nasrawin, 2023; Vahedian-Azimi & Moayed, 2021). These studies support the finding that higher self-esteem is associated with higher motivation, helping improve academic performance in diverse student populations and educational levels.

Finally, it was found that student personality is directly related to student motivation and anxiety, so motivation and anxiety levels in university students vary according to their characteristics. The results are consistent with those obtained in the Farahati et al. (2014), Kaur et al. (2022) and Ke (2024) studies, which showed how motivation and anxiety varied significantly according to the personality types of the individuals analyzed. These results seem to suggest that more motivated students procrastinate less in their academic tasks, causing low anxiety levels, so depending on their personality traits and motivation levels, we can predict how likely they are to engage in procrastination behavior. Understanding the connection between personality types and students’ anxiety and motivation can lead to more personalized and effective treatment approaches.

To answer the third research question of the study and determine whether it is possible to predict the level of procrastination perceived by students by knowing the dimensions associated with procrastination, a linear regression study was conducted. The regression results show that the proposed model is significant in predicting the level of perceived procrastination at 30%. These data indicate that the model could predict the probability of procrastination in 1 out of 3 students. Perceived procrastination is influenced by several factors, with screen time as a positive and significant predictor. These results align with previous studies suggesting that increased use of technology by students is associated with multitasking, procrastination, and poor time management (Mesfin & Gebremeskel, 2023). On the other hand, positive self-regulation was confirmed as a protective factor against perceived procrastination, while negative self-regulation acts as a risk factor. This data reinforces that students’ self-regulation directly influences their study and time management habits by affecting their procrastination patterns and academic success (Lourenço & Paiva, 2024). Finally, self-efficacy also significantly influences perceived procrastination, with a positive effect. This information suggests that higher levels of self-efficacy are associated with greater procrastination, an unexpected result. We obtain a negative or inverse effect of this dimension by analyzing the effect of self-efficacy in isolation as a factor in determining perceived procrastination. However, when we add the dimension of self-regulation, this relationship between self-efficacy and perceived procrastination changes its sign, as shown in the results. These data suggest that positive self-regulation is a mediating or suppressing factor in the relationship between self-efficacy and procrastination. When introduced into the model, much of the effect initially attributed to self-efficacy is redistributed, revealing that positive self-regulation has a significant impact on reducing procrastination. Self-efficacy, being highly correlated with self-regulation, loses explanatory power when the latter is controlled for, and its effect even changes sign, indicating that it is not a key predictor. These results highlight the importance of students’ awareness of screen time and their levels of self-regulation and self-efficacy in identifying their procrastination habits. With good self-regulation and self-efficacy mechanisms, students who control their screen time will have a lower perception of procrastination, allowing them to adopt more effective strategies to manage their time and improve their academic performance.

The studies presented highlight how excessive use and abuse of social media and prolonged exposure to screens can significantly contribute to the increase in procrastination among students in academic settings. This phenomenon is worrying in a context where researchers are increasingly aware of aspects such as motivation, mental health, and anxiety, which are negatively affected by the massive use of social media and excessive screen consumption among young people. However, these data are considered preliminary and inconclusive and need further exploration, with more extensive studies examining these aspects in depth and a larger sample size.

It is, therefore, imperative to highlight the importance of addressing these dimensions in the educational environment. It is essential to develop strategies that assess and predict the negative impact of these behaviors on students’ academic performance and to implement effective intervention programs. These programs should be designed to educate and encourage a balanced and healthy use of electronic devices. It is also interesting to include components that address self-regulation, self-efficacy, and self-esteem in such programs, as these are crucial in mitigating the adverse effects of excessive use of electronic devices. Measuring the impact of these strategies is equally crucial, and evaluation tools should be used to monitor students’ progress and adjust programs as necessary. These programs will not only help improve academic performance but also contribute to students’ psychological well-being, reducing anxiety symptoms, improving overall mental health, and better preparing them to face the challenges of today’s digital world.

## 5. Conclusions

This study investigated the joint influence of screen use and key psychological dimensions on academic procrastination among college students, providing a predictive model that explains a substantial portion of the variance in perceived procrastination. The model reveals that screen time, self-regulation, and self-efficacy are significant predictors, suggesting that approximately one in three cases of procrastination can be predicted based on this combination of behavioural and psychological factors.

The questionnaire developed for this research, although it still requires a complete validation process, has been constructed from existing validated scales in the literature and demonstrates adequate internal consistency, enabling the simultaneous assessment of screen use habits and multiple dimensions related to procrastination. These results make it a useful screening tool for preliminary diagnosis in institutional surveys or prevention programs, as it helps professionals detect patterns of high screen use associated with low self-regulation, low self-efficacy and self-esteem, along with high anxiety.

Students show high screen use, although in general, they do not exhibit highly problematic addictive behaviours. However, evidence suggests that around 20% of students may have critical levels of screen use, underscoring the need for universities to incorporate digital well-being and time management components into their curricula and counselling services. In turn, high screen use is associated with lower levels of self-regulation, self-efficacy, and self-esteem, and higher levels of anxiety. On the other hand, high levels of self-regulation and self-efficacy are related to more motivated students with better self-esteem and fewer signs of anxiety. For teaching staff and student support services, these results highlight that interventions aimed at improving self-regulation and self-efficacy can have a double benefit: they can reduce levels of procrastination while promoting better emotional adjustment and greater academic engagement.

Screen time, students’ self-regulation strategies, and self-efficacy predict perceived procrastination behaviours among college students. The regression model developed here can serve as an empirical basis for monitoring risk over time and evaluating the impact of interventions, for example, by tracking whether changes in self-regulation strategies or self-efficacy beliefs accompany reductions in perceived procrastination. In this sense, the research offers concrete and applicable indicators that can serve as a basis for institutional policies, psychoeducational workshops, and individual counselling.

From the perspective of the study’s limitations, extending this work to larger and more diverse samples, including multiple universities and countries, and utilising longitudinal and more advanced analytical approaches (e.g., confirmatory factor analysis and structural equation modelling) would strengthen the generalizability and causal interpretation of the results. Furthermore, as this was a cross-sectional study, no formal control group was included. Therefore, the results should be interpreted as correlational patterns within the study population, rather than as causal effects that are comparable across different risk groups.

Although the model analysed is significant and explains a moderate proportion of the variability in procrastination, the unexplained part of the variance suggests that future models should incorporate additional factors, such as contextual variables, teaching practices, or broader personality traits. Nevertheless, the present study already provides a solid starting point. It demonstrates the potential of integrating screen use metrics with the psychological dimensions of procrastination to guide evidence-based decision-making in higher education.

Further efforts can identify and analyse the profiles or patterns of electronic device use among university students to explore their relationship with academic procrastination. This line of research could provide clues to understand better how certain technological habits, which differentiate groups of students according to variables such as frequency, type of device, or most frequently used applications, are related to procrastination behaviours. Understanding these dynamics could provide valuable information for designing more specific and effective interventions to reduce procrastination behaviours associated with problematic or unplanned use of electronic devices.

## Figures and Tables

**Table 1:** Sample used for each RQ in the study.

Sample	RQ	Study
Sample 1: 924	RQ3	Predictive regression models
Subsample or Sample 2: 386	RQ1 RQ2	Screen-use patterns and their association with procrastination

**Table 2:** Scales used per dimension.

Dimensions	Scales	Cronbach’s Alpha	Authors (Year)
Screens	Adolescent Preoccupation with screens scale (APSS)	Emotional = 0.91 Behavioural = 0.87	Hunter et al. (2017)
	Social Media Disorder Scale (9-item SMD scale)	0.76	van den Eijnden et al. (2016)
Self-regulation	(ASRS) Academic Self-Regulation Scale	0.83	Akhtar and Mahmood (2013)
	(EMSR-Q) Emotion and motivation Self-Regulation Questionnaire	Avoidance = 0.69 Performance = 0.72 Negative = 0.79 Positive = 0.70 Process = 0.70	Alonso-Tapia et al. (2014)
Self-efficacy	Student Self-Efficacy (SSE)	0.84	Rowbotham and Schmitz (2013)
Motivation	Academic Motivation Scale-College (AMS-C)	0.81	Vallerand et al. (1992)
Self-esteem	Rosenberg Self-Esteem Scale	0.84	Rosenberg (1965)
Personality	(IPIP) International Personality Item Pool	Extroversion = 0.87 Agreeableness = 0.82 Conscientiousness = 0.79 Neuroticism = 0.86 Openness to Experience = 0.84	Goldberg et al. (2006)
Anxiety	TAI-5 item: Test Anxiety Inventory	0.87	Taylor and Deane (2002)
	(STAI-T) The State-Trait Anxiety Inventory	Trait = 0.90 State = 0.94	Spielberger (1970)

**Table 3:** Reliability coefficients per scale.

Scale	No. Item	α-Cronbach
Screen Use	18	0.892
Self-Regulation	10	0.614
Self-Efficacy	10	0.892
Motivation	12	0.848
Self-Esteem	10	0.899
Personality	10	0.651
Anxiety	10	0.883
Total (N = 386)	80	0.852

**Table 4:** Descriptive statistics for the dimensions of procrastination.

Dimensions	N	Minimum	Maximum	Mean	Standard Dev.
Self-Regulation	386	1.90	4.8	3.286	0.48162
Self-Efficacy	386	1.00	5.0	4.0106	0.63509
Motivation	386	1.91	5.0	4.1003	0.65138
Self-Esteem	386	1.60	5.0	3.6549	0.76866
Anxiety	386	1.00	5.0	3.2826	0.8572
Personality	386	1.00	4.9	3.6466	0.51427

1 = Never, 2 = Almost never, 3 = Sometimes, 4 = Almost always, 5 = Always.

**Table 5:** Correlation of study dimensions.

		Self-Efficacy	Personality	Anxiety	Self-Regulation	Motivation	Self-Esteem
SCREENS	Pearson correlation	−0.205 **	0.095	0.375 **	−0.276 **	0.02	−0.313 **
	Sig. (bilateral)	0	0.063	0	0	0.698	0
SELF_EFFICACY	Pearson correlation	1	0.204 **	−0.291 **	0.334 **	0.422 **	0.528 **
	Sig. (bilateral)		0	0	0	0	0
PERSONALITY	Pearson correlation		1	0.336 **	0.246 **	0.343 **	0.022
	Sig. (bilateral)			0	0	0	0.667
ANXIETY	Pearson correlation			1	−0.134 **	0.084	−0.555 **
	Sig. (bilateral)				0.008	0.101	0
SELF-REGULATION	Pearson correlation				1	0.429 **	0.328 **
	Sig. (bilateral)					0	0
MOTIVATION	Pearson correlation					1	0.204 **
	Sig. (bilateral)						0

**. The correlation is significant at the 0.01 level (bilateral).

**Table 6:** Regression Model Summary.

Model	R	R Square	R Square Corrected	Standard Error of Estimation	Durbin- Watson
1	0.564	0.318	0.310	0.67899	2.048

**Table 7:** Analysis of Variance (ANOVA) of Model.

Model		Sum of Squares	gf	Quadratic Mean	F	Sig.
1	Regression	195.927	11	17.812	38.634	0.000
	Residual	420.463	912	0.461		
	Total	616.390	923			

**Table 8:** Linear Regression Coefficients.

Model	Unstandardised Coefficients		Typified Coefficients	t	Sig.
	B	Standard Error	Beta		
(Constant)	2.744	0.342		8.021	0.000
Screens_TimeUse	0.302	0.039	0.277	7.790	0.000
Screens_Emotions	−0.037	0.045	−0.030	−0.828	0.408
Screens_Problems	−0.012	0.040	−0.010	−0.293	0.770
SelfRegulation_Positive	−0.484	0.038	−0.432	−12.704	0.000
SelfRegulation_Negative	0.149	0.043	0.115	3.495	0.000
SelfRegulation_Class Attendance	0.013	0.033	0.012	0.406	0.685
Self_Efficacy	0.151	0.045	0.112	3.341	0.001
Motivation	0.015	0.055	0.008	0.271	0.786
Self_Esteem	0.044	0.074	0.018	0.603	0.547
Personality	0.050	0.054	0.029	0.927	0.354
Anxiety	−0.016	0.034	−0.016	−0.454	0.650

## Data Availability

The original contributions presented in this study are included in the article. Further inquiries can be directed to the corresponding author.
